# Improved Innate and Adaptive Immunostimulation by Genetically Modified HIV-1 Protein Expressing NYVAC Vectors

**DOI:** 10.1371/journal.pone.0016819

**Published:** 2011-02-15

**Authors:** Esther D. Quakkelaar, Anke Redeker, Elias K. Haddad, Alexandre Harari, Stella Mayo McCaughey, Thomas Duhen, Abdelali Filali-Mouhim, Jean-Philippe Goulet, Nikki M. Loof, Ferry Ossendorp, Beatriz Perdiguero, Paul Heinen, Carmen E. Gomez, Karen V. Kibler, David M. Koelle, Rafick P. Sékaly, Federica Sallusto, Antonio Lanzavecchia, Giuseppe Pantaleo, Mariano Esteban, Jim Tartaglia, Bertram L. Jacobs, Cornelis J. M. Melief

**Affiliations:** 1 Department of Immunohematology and Blood Transfusion, Leiden University Medical Center, Leiden, The Netherlands; 2 Laboratoire d'Immunologie, Centre de Recherche du Centre Hospitalier de l'Université de Montréal (CR-CHUM), Montreal, Canada; 3 Division of Immunology and Allergy, Centre Hospitalier Universitaire Vaudois, Lausanne, Switzerland; 4 Swiss Vaccine Research Institute, Lausanne, Switzerland; 5 Department of Medicine, University of Washington, Seattle, Washington, United States of America; 6 Institute for Research in Biomedicine, Bellinzona, Switzerland; 7 Centro Nacional de Biotecnologia, CSIC, Madrid, Spain; 8 Arizona State University, Tempe, Arizona, United States of America; 9 Sanofi Pasteur, Swiftwater, Pennsylvania, United States of America; 10 ISA Pharmaceuticals B.V., Bilthoven, The Netherlands; 11 Vaccine and Infectious Diseases Division, Fred Hutchinson Cancer Research Center, Seattle, Washington, United States of America; University of Cape Town, South Africa

## Abstract

Attenuated poxviruses are safe and capable of expressing foreign antigens. Poxviruses are applied in veterinary vaccination and explored as candidate vaccines for humans. However, poxviruses express multiple genes encoding proteins that interfere with components of the innate and adaptive immune response. This manuscript describes two strategies aimed to improve the immunogenicity of the highly attenuated, host-range restricted poxvirus NYVAC: deletion of the viral gene encoding type-I interferon-binding protein and development of attenuated replication-competent NYVAC. We evaluated these newly generated NYVAC mutants, encoding HIV-1 *env*, *gag*, *pol* and *nef*, for their ability to stimulate HIV-specific CD8 T-cell responses *in vitro* from blood mononuclear cells of HIV-infected subjects. The new vectors were evaluated and compared to the parental NYVAC vector in dendritic cells (DCs), RNA expression arrays, HIV *gag* expression and cross-presentation assays *in vitro*. Deletion of type-I interferon-binding protein enhanced expression of interferon and interferon-induced genes in DCs, and increased maturation of infected DCs. Restoration of replication competence induced activation of pathways involving antigen processing and presentation. Also, replication-competent NYVAC showed increased Gag expression in infected cells, permitting enhanced cross-presentation to HIV-specific CD8 T cells and proliferation of HIV-specific memory CD8 T-cells *in vitro*. The recombinant NYVAC combining both modifications induced interferon-induced genes and genes involved in antigen processing and presentation, as well as increased Gag expression. This combined replication-competent NYVAC is a promising candidate for the next generation of HIV vaccines.

## Introduction

Development of an effective HIV-1 vaccine inducing both broadly neutralizing antibodies and virus-specific T cells has the best chance to inhibit HIV-1 replication, infection and acquisition. However, the design of vaccines that can do both has been extremely difficult [1]–[4]. The correlation between HIV-specific CD8 T-cell responses and control of viral load as well as the correlation between certain HLA-types and slow disease progression [5]–[13] underscore that T cells could limit the extent of subsequent viral replication. As a result, potent vaccine-induced HIV-1-specific T-cell responses could decrease tissue damage during the acute phase of infection and improve the control of virus replication leading to a lower viral load set point, thus reducing viral transmission and delaying progression to AIDS. A vaccine that is able to induce robust long lasting T-cell responses is, therefore, likely to have an impact on the HIV-1 epidemic.

The highly-attenuated vaccinia virus strain NYVAC is under intense preclinical and clinical investigation due to its efficacy and safety as a recombinant vaccine against multiple diseases [14]–[21]. The NYVAC strain was derived from the Copenhagen vaccinia strain. Deletion of 18 open reading frames (ORFs) implicated in the pathogenicity and virulence of *Orthopoxviruses*, as well as in host-range regulatory functions involving the replication competence of these viruses, resulted in its attenuated phenotype [22]. The high level of attenuation of this vector is illustrated by its failure to spread in immunodeficient mice, its dramatically reduced ability to replicate in a variety of human cells in tissue culture, and its inability to produce infectious virus in human beings [22]. Despite its limited replication in most mammalian cell types, it provides a high level of gene expression and triggers strong immune responses when delivering foreign antigens in animals and human beings [17], [18], [21], [23]–[27]. These beneficial effects have stimulated the use of the NYVAC vector for vaccination against HIV and other infectious diseases [15], [28].

NYVAC expressing SIV or HIV-1 antigens (*env, gag, pol or nef*) has been the subject of several preclinical and clinical studies. Protection from disease progression and control of viral load has been observed in macaques immunized with NYVAC expressing *env* (gp120) of SHIV_89.6P_ and *gag-pol-nef* of SIV_mac239_ or *gag-pol-env* of SIV_mac251_, subsequently challenged with pathogenic SHIV_89.6P_ or SIV_mac251_ [25], [26], [29], [30]. A phase I clinical study showed that the combination of DNA/NYVAC expressing *env* (gp120)-*gag*-*pol*-*nef* of HIV-1 from clade C triggered antigen specific immune responses in 90% of volunteers with maintenance of these responses for at least 72 weeks [19], [20]. Despite these promising immunogenicity data, the response was mainly directed to *env* and the T cells were predominantly CD4+ [25]. Thus, improvement of the NYVAC vector is necessary to further enhance the strength and breadth of HIV-specific T-cell responses [31]. The recently published results from the Thai trial, in which a moderate protective effect of the recombinant canary poxvirus ALVAC in combination with protein gp120 has been described [32], underscores the need for improvement, while simultaneously showing protective potential.

To improve immunogenicity of the NYVAC vector we followed two strategies. First, the B19R viral gene encoding a soluble protein preventing binding of type-I interferon (IFN) to its natural receptor [33]–[37] was deleted (Kibler et al., submitted for publication). Second, the replication capacity of NYVAC was restored by inserting two viral host range genes, K1L and C7L [31], [38]–[41], resulting in a replication-competent but attenuated NYVAC vector (Kibler et al., submitted for publication). Here, we have performed an in-depth characterization of the biological responses of the parental NYVAC virus and its recombinant mutants in human cells *in vitro*.

Our findings reveal marked differences among the replication-competent vectors, gene deletion vector, and unmodified NYVAC. Deletion of the B19R IFN-binding protein resulted in enhanced expression of IFN and IFN-induced genes, transcription factors and target genes, both in conventional and plasmacytoid DCs. In conventional DCs, this was associated with IFN-α production and enhanced expression of the co-stimulatory molecule CD86. Restoration of replication competence activated pathways involved in processing and presentation of HIV and poxvirus antigens to T cells. Combination of the two strategies resulted in the expression of pathways enriched in both IFN-induced genes and antigen processing. Indeed, replication-competent NYVAC showed substantially increased expression of Gag in the infected target cells, permitting significant improvement in cross-presentation to HIV-specific T cells as well as enhanced induction of HIV-specific memory CD8 T-cell responses *in vitro*.

## Results

### Enhanced IFN-α production by cDCs after infection with NYVAC lacking the type-I IFN-binding protein

**Figure 1:**
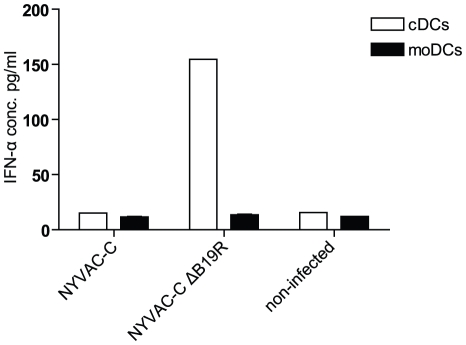
IFN-α production after infection of DCs with recombinant NYVAC. cDCs (white bars), and moDCs (black bars) were infected for one hour with the two different recombinant viruses NYVAC-C and NYVAC-C-ΔB19R (MOI 1). Forty-eight hours post infection, IFN-α production was analyzed by ELISA. Mean values of two independent experiments are shown. Deletion of type-I IFN binding protein resulted in enhanced IFN-α production after infection of cDCs.

To improve immunogenicity of the attenuated NYVAC strain, we first generated a virus that lacks the gene coding for a soluble protein preventing binding of type-I IFN to its natural receptor (Table 1). This NYVAC-C-ΔB19R has been analyzed for its effect on monocyte-derived and conventional DCs. NYVAC-C-ΔB19R infected cDCs produced IFN-α 48 hours post-infection, whereas NYVAC-C infected cDCs did not (figure 1). In contrast, moDCs did not produce IFN-α after infection with either virus. pDCs infected with both NYVAC-C or NYVAC-C-ΔB19R resulted in high IFN-α production (>400 pg/ml; data not shown).

In conclusion, deletion of the type-I IFN-binding protein B19R resulted in enhanced IFN-α production in cDCs.

**Table 1:** Nomenclature and description of the generated NYVAC viruses.

Full name	Description	Ref.
NYVAC-C	Attenuated Copenhagen strain of vaccinia virus containing HIV clade C *gag*, *pol*, *nef* and *env* genes	[17], [22]
NYVAC-C-ΔB19R	Deletion of B19R gene that encodes for a type I IFN receptor homologue in the background of NYVAC-C	1 [33]
NYVAC-C-K1L-C7L (NYVAC-C-KC)	Host restriction genes K1L and C7L have been reinserted in NYVAC genome to restore replication competence	1 [40]
NYVAC-C-K1L-C7L-ΔB19R (NYVAC-C-KC-ΔB19R)	Deletion of B19R gene that encodes for a type I IFN receptor homologue in the background of the replication-competent NYVAC	1 [33]

Simplified nomenclature of the viruses is indicated between brackets.

1 Kibler et al. Submitted for publication.

### Restored replication competence of NYVAC in human cells

**Figure 2:**
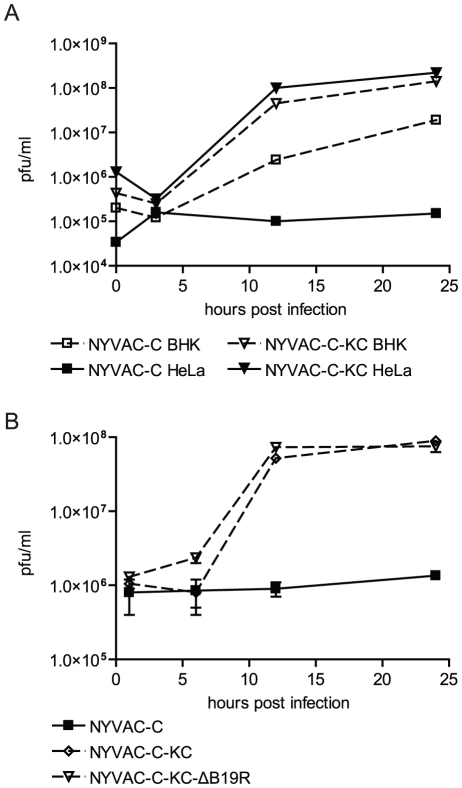
Replication of NYVAC-C and NYVAC-C-KC in human HeLa cells. A) Human HeLa cells (solid line, closed symbols) or BHK cells (dashed line, open symbols) were infected with NYVAC-C (triangles) or NYVAC-C-KC (squares). B) HeLa cells were infected with NYVAC-C (black squares) or the replication-competent NYVAC-C-KC (open diamond) and NYVAC-C-KC-ΔB19R (open triangle). A MOI of 5 was used for all infections. Cultures were harvested immediately after infection, or at the indicated time points post infection. Virus was released from cells by multiple rounds of freezing and thawing, and released virus was titrated on permissive BHK cells. Introduction of K1L and C7L into NYVAC-C fully restored replication competence in human HeLa cells, comparable to replication in BHK cells. Additional deletion of the B19R gene did not alter replication capacity. Data representative of at least 3 independent experiments are shown.

We also generated a virus mutant with reintroduced genes restoring virus replication competence (Table 1). To investigate whether reinsertion of the K1L and C7L genes, involved in virus host range restriction [39]–[41], resulted in increased replication capacity in human cells, we determined viral replication of NYVAC-C and NYVAC-C-KC in human (HeLa) and hamster (BHK) cells (figure 2A). Replication capacity is represented by the increasing virus titers recovered at different time points after infection. Both vectors were fully replication competent in BHK cells, but replication of NYVAC-C was restricted in HeLa cells. Reinsertion of K1L and C7L in NYVAC slightly increased replication in BHK and fully restored replication in HeLa cells. Additional deletion of the B19R gene did not influence replication capacity of NYVAC-C-KC in human HeLa cells (figure 2B). These data show that the replication capacity of NYVAC in HeLa cells was restored by the insertion of two ORFs, K1L and C7L.

### NYVAC-C-KC, NYVAC-C-ΔB19R and NYVAC-C-KC-ΔB19R induced expression of common and unique genes in infected DCs

**Figure 3:**
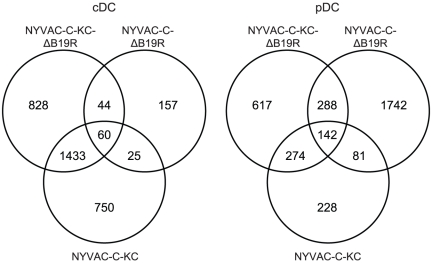
Venn diagram of the number of common and unique genes in cDCs and pDCs after infection with NYVAC-C and its mutants. Venn-diagrams showing the numbers of genes that are up- and down-regulated in cDCs (left panel) and pDCs (right panel) after infection with NYVAC-C-ΔB19R, NYVAC-C-KC or NYVAC-C-KC-ΔB19R. In cDCs 157, 750 and 828 genes are uniquely differentially expressed (p<0.05) in NYVAC-C-ΔB19R, NYVAC-C-KC and NYVAC-C-KC-ΔB19R, respectively. In pDCs 1742, 228 and 617 genes are uniquely differentially expressed (p<0.05) in NYVAC-C-ΔB19R, NYVAC-C-KC and NYVAC-C-KC-ΔB19R, respectively. For each gene, the expression induced by NYVAC-C-ΔB19R, NYVAC-C-KC or NYVAC-C-KC-ΔB19R was tested for differential expression by comparison to the expression induced by NYVAC-C (n ranges between 2 and 18).

We next sought to determine the global transcriptional signature of the different poxviruses in *ex vivo* derived cDCs and pDCs. Sorted cDCs and pDCs were either infected with NYVAC-C-ΔB19R, NYVAC-C-KC or NYVAC-C-KC-ΔB19R. RNA was extracted and processed for gene array analysis. Figure 3 shows two Venn diagrams for cDCs (left) and pDCs (right) demonstrating the number of common and unique differentially expressed genes, induced by the three poxviruses, in the two DC subsets. These Venn diagrams were obtained by comparing the list of differentially expressed genes between each poxviruses and NYVAC-C group samples. For example, NYVAC-KC-ΔB19R induced 828 and 617 unique genes in cDCs and pDCs, whereas NYVAC-C-KC induced 750 and 228 unique genes in the corresponding DC subsets. These diagrams also show that the different poxviruses induced common genes in the DC subsets; NYVAC-C-KC and NYVAC-C-KC-ΔB19R induced 1433 and 274 common genes in cDCs and pDCs, respectively. These genes were significantly up or down regulated (p-value<0.05). The lists of the unique genes for each mutant are presented in table S1, S2 and S3 for cDCs and S4, S5 and S6 for pDCs. A list of all common genes between all three mutants is represented in table S7.

These results indicate that different poxviruses have the ability to elicit distinct and common genes in DCs and that poxvirus with multiple mutations induced distinct transcriptional profiles in cDCs and pDCs that were different from those induced by single mutants.

### Combination of the B19R deletion and replication competence resulted in expression of pathways targeted by both single mutants

We performed gene set enrichment analysis (GSEA) [42] to identify the pathways that are differentially expressed in cDCs and pDCs infected with different NYVAC mutants. GSEA was performed by interrogating three GSEA molecular signatures databases, namely the C2, C3 and C5 and a collection of 28 immune related gene sets described by Chaussabel *et al*. [43]. As expected, NYVAC-C-ΔB19R induced the enhanced expression of genes in the type-I IFN-induced gene pathways and IL-1R (inflammasome) in pDCs (figure S1A). These pathways include genes like NFκB1, IFN-α, TRAFD and many others (figure S1A). A representative list with genes of each of the pathways is depicted in the right vertical line. We also observed increased expression of genes encoding target molecules for the transcription factors IRF1, IRF2, IRF7 and other IFN-inducible transcription factors (figure S1B). Similar pathways were induced in NYVAC-C-ΔB19R infected cDCs (figure S1D-E). These results indicate that NYVAC-C-ΔB19R induced the expression of IFN-induced pathways and IFN-regulated transcription factors as well as multiple inflammatory cytokines.

NYVAC-C-KC elicited the induction of pathways associated with antigen processing and presentation as well as of genes involved in B-cell help in cDCs (figure S2A). No expression of IFN-induced genes and inflammatory pathways was observed. A representative list with genes of each of the pathways is depicted in the right vertical line. For example, the antigen processing and presentation pathway includes genes of HLA, TAPBP, CIITA, TAP1, and TAP2, CD40, ICAM1, and ICOSL are genes included in the B-cell function pathway (see table S8 for a complete list of genes of the corresponding pathways). NYVAC-C-KC induces distinct gene set enrichment pathways in pDCs (figure S2C–D) compared to cDCs (figure S2A–B). Differences in gene expression are less clear between NYVAC-C and NYVAC-C-KC in infected pDCs.

**Figure 4:**
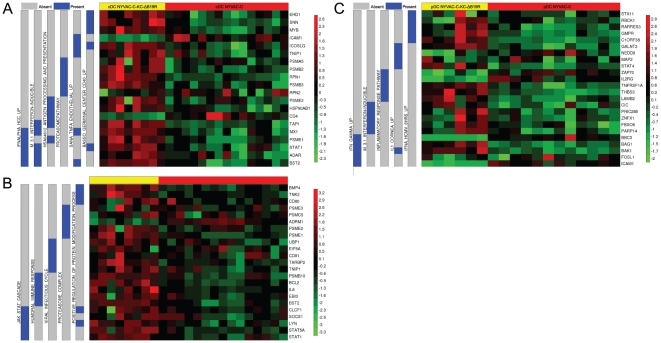
Gene set enrichment analysis of NYVAC-C and NYVAC-C-KC-ΔB19R infected cDCs and pDCs. GSEA of the list of genes ranked according to the expression difference between NYVAC-C and NYVAC-C-KC-ΔB19R in cDCs (A-B) and pDCs (C). GSEA using C2 database (A, C) and C5 database (B) is shown. Figure shows the pattern of enrichment using selected significant pathways and their top 5 genes members selected from the leading edge subset (genes that contribute most to the enrichment score). The left gray and blue section of the figure shows the pathway membership for each gene (blue, present in the pathway; grey, absent). The heatmap shows the expression level of each gene scaled to have mean zero and standard deviation one (red, up-regulated; green, down-regulated). Each column in the heatmap represents a replicate (n ranges between 6 and 15). The color key is depicted on the right side of the figure. The NYVAC-C-KC-ΔB19R mutant induced the expression of IFN genes in cDCs and pDCs, as well as genes involved with antigen processing and presentation genes.

Introduction of both the B19R mutation and replication competence into NYVAC-C enriched pathways specific for both mutants. The NYVAC-C-KC-ΔB19R mutant induced the expression of IFN genes, as well as genes involved with antigen processing and presentation genes including the proteasome pathway (figure 4).

Overall, NYVAC-C-ΔB19R induced the expression of IFN and IFN-induced genes, transcription factors and target genes, both in cDCs and pDCs. A summary of the genes that contribute to the enrichment of the IFN signaling and germinal center pathways is provided in table 2. Restoration of replication competence in NYVAC-C-KC induced distinct signaling pathways in cDCs and pDCs. NYVAC-C-KC activated pathways that enhance processing and presentation of antigens to T cells in cDCs. Combination of these two strategies represented by the NYVAC-C-KC-ΔB19R mutant resulted in the expression of pathways enriched in IFN-induced genes and antigen processing and presentation (table 2). These genes are important in innate and adaptive immunity and have the potential to improve cell-mediated immune responses.

**Table 2:** Summary of genes that contribute to the enrichment of the interferon signaling or germinal center pathway after infection with the indicated recombinant NYVAC compared to NYVAC-C.

Gene	NYVAC-C-dB19R				NYVAC-C-KC				NYVAC-C-KC-dB19R			
	Interferon Signaling	Germinal Center (CD40) 1	Interferon Signaling	Germinal Center (CD40)^1^	Interferon Signaling 2	Germinal Center (CD40)	Interferon Signaling 2	Germinal Center (CD40)	Interferon Signaling	Germinal Center (CD40)^3^	Interferon Signaling	Germinal Center (CD40)^3^
	pDC		cDC		pDC		cDC		pDC		cDC	
SERPING1	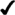								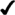		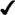	
TRAFD1	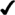		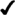						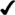		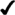	
EIF2AK2	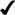		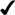						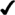		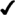	
OASL	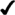		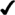						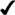			
STAT1	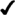	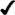	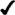	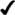				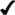	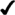	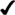	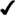	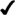
ADAR	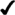		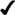						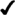		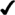	
IFITM2	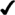								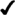			
IRF7	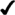		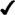						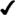		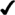	
MX1			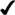								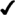	
IRF1			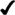								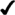	
CXCL10	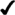		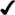						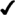			
BCL2								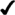			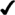	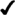
MYB		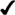						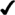		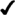		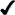
ICOSLG						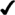		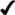				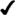
STAT5A						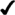		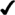				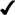
CD40						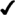		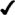				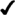
TNF						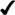		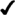				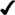
CCR7						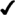		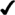		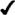		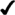
LYN		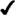		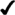				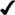				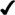

Marked are the genes that contribute to the enrichment of a given pathway, for a given subset.

1 Germinal Center pathways are not significantly regulated with NYVAC-C-dB19R.

2 Interferon is not significant in both pDCs and cDCs for NYVAC-C-KC vs NYVAC-C.

3 Germinal Center pathways are significantly regulated within cDC NYVAC-C-KC-dB19R and not in pDCs.

### Gene set enrichment analysis revealed the induction of distinct signaling pathways in response to recombinant NYVAC

The above-described results focused on pathways expected to be targeted by the B19R deletion and restoration of replication competence. However, gene expression arrays allowed for an exploration of all genes up- or down-regulated in infected DCs. In addition to IFN-induced genes, NYVAC-C-ΔB19R induced the enhanced expression of genes involved in TOLL like receptor signaling and JAK/STAT pathways (figure S1A). We also observed the induction of genes associated with cytokine activity, immune effector functions, and IKB kinase activity (figure S1C). NYVAC-C-KC induced pathways with genes involved in cellular activation, cell adhesion and germinal center activation (figure S2A). Representative genes of each of these pathways are depicted in the right vertical line. Furthermore, NYVAC-C-KC also induced increased expression in target genes downstream of the transcription factors Sp3, POU3F2, CREL, TEF1 and E2F. For example, FOXP1 and GADD45G are genes downstream of the transcription factor Sp3; SOX4 and HOXA11 are target genes for POU3F2; MSC and EHD1 target genes for CREL; ATP1B1 and CYP26A1 target genes for TEF1; and RAD51 and YWHAQ target genes for E2F. A comprehensive list of genes downstream of these transcription factors is present in figure S2B. NYVAC-C-KC induced distinct pathways in cDCs as compared to pDCs, shown in figure S2B. pDCs infected with NYVAC-C-KC expressed genes associated with inflammation, IL-6 induction, and Wnt pathways (figure S2C). We also observed increased expression in target genes of the transcription factors NFAT, CEBP and STAT5A (figure S2D). Gene set enrichment analysis of NYVAC-C-KC-ΔB19R infected cDCs also showed expression of genes involved in B-cell help, TNF-α and proteasome pathways (figure 4A). Together, these induced pathways have the potential to improve immunogenicity of NYVAC as HIV vaccine.

### Increased CD86 expression in DCs infected with NYVAC-C-ΔB19R

**Figure 5:**
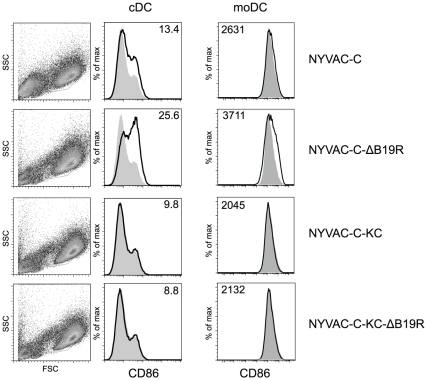
Maturation of cDCs after infection with recombinant NYVAC. Expression of CD86 on infected cDCs and moDCs is shown. DCs were infected for one hour with the different recombinant vectors and their phenotype was analyzed by flow cytometry after 48 hours of culture. The shaded graphs represent NYVAC-wt infected DCs; solid lines represent the indicated recombinant NYVAC. Mean fluorescence intensity (MFI) is indicated in the plots. MoDC and cDC infected with NYVAC-C-ΔB19R showed enhanced CD86 expression in contrast to DCs infected with the parental NYVAC-C or NYVAC-C-KC. Infection with NYVAC-C-KC-ΔB19R did not induce increased CD86 expression. Data are representative of at least two independent experiments.

In addition to the gene expression arrays, we also studied the impact of infection with recombinant NYVAC mutants on the maturation of DCs. Both cDCs and moDCs infected with NYVAC-C-ΔB19R showed increased CD86 expression 48 hours after infection (figure 5). In contrast, NYVAC-C-KC did not mature cDCs or moDCs at all. Expression levels of CD86 were even lower compared to NYVAC-C infected DCs. Of note, combination of replication competence with the B19R gene deletion did not permit enhanced maturation of the infected DCs. In contrast to cDCs and moDCs, pDCs did not mature after infection (data not shown).

In conclusion, the enhanced maturation of the infected cells following infection of cDCs with the NYVAC vector lacking the type-I IFN decoy receptor, B19R, was only observed in the background of the non-replicating vector, but not in combination with replication-competent NYVAC-C-KC. Although several pathways important for induction of immune responses showed enhanced activity at RNA level after NYVAC-C-KC-ΔB19R infection, the level of activity was lower than in the NYVAC-C-ΔB19R infected DCs (figure 4 versus S2).

### Enhanced HIV-1 Gag expression by replication-competent NYVAC in human cells

**Figure 6:**
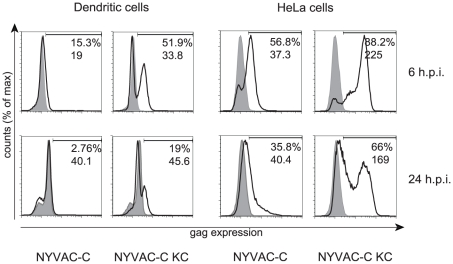
Gag expression in human moDCs and HeLa cells. Histograms show α-Gag KC57 staining in infected moDCs and HeLa cells. Cells were infected at MOI 5 for one hour. After six and 24 hours incubation, cells were harvested and stained for Gag expression by ICS as described in the Materials and Methods. Percentage of Gag-expressing cells and median fluorescence intensity were determined and indicated in the graphs. Shaded graphs represent staining of NYVAC-wt infected cells. Solid line represents cells infected with the different variants. Gag expression after infection with NYVAC-C-KC is higher compared to NYVAC-C, both in moDC and HeLa cells, at multiple time points after infection, correlating with the increased replication capacity of NYVAC-C-KC. Data are representative of at least three similar independent experiments.

The restored replication competence of NYVAC-C-KC is expected to increase transgene expression in infected cells. To study the HIV-antigen expression, HeLa cells and human moDCs were infected and Gag expression was determined by flow cytometry at 6 and 24 hours post infection (figure 6). Cells infected with NYVAC-C-KC showed substantially higher percentages of infected cells, as well as higher median fluorescence intensity of the Gag expressing cells compared to cells infected with NYVAC-C. Both HeLa and moDCs infected with NYVAC-C showed a reduction in the percentage of Gag expressing cells at 24 hours post infection. The FSC/SSC plots and propidium iodide staining (not shown) suggest the presence of apoptotic or necrotic cells at 24 hours post infection. Although we cannot discriminate between apoptosis and necrosis in our assays, induction of apoptosis in HeLa cells after infection with NYVAC has been described before [44]. The percentage of NYVAC-C-KC infected cells expressing Gag was also decreased at 24 hours post infection, though there was considerably more Gag expression compared to that in cells infected with NYVAC-C. Deletion of the B19R gene did not influence Gag expression by either virus vector (data not shown). Of note, we observed that Gag expression in moDCs and the percentage of gag expressing cells were lower when compared to HeLa cells. In both moDCs and HeLa cells, clear differences were observed in Gag expression between the host-range restricted NYVAC-C and the replication-competent NYVAC-C-KC.

The increased median fluorescence intensity of NYVAC-C-KC compared to NYVAC-C (225 vs. 37.3 and 169 vs. 40.4 at 6 and 24 hours post infection, respectively; figure 6) reflects increased Gag expression in HeLa cells. This fully correlates with the viral gene expression patterns of the two viruses; NYVAC gene expression in HeLa cells is restricted at later times [38], whereas in HeLa cells infected with the replication-competent vector late products are made and viral progeny is produced. This virus replication cycle is reflected in the far right peak observed in HeLa cells (figure 6, right panels).

### Improved cross-presentation of replication-competent NYVAC

**Figure 7:**
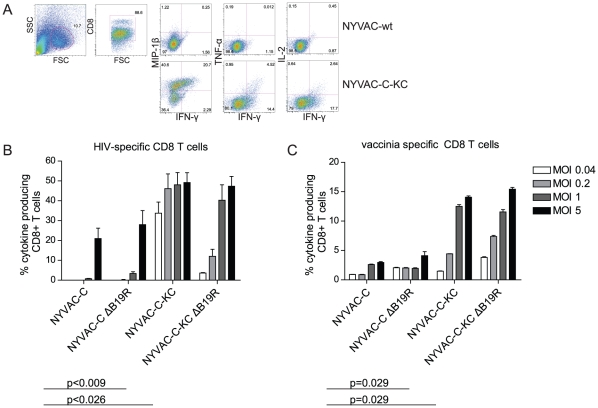
Antigen cross-presentation to HIV- and vaccinia-specific CD8 T-cell clones. MoDCs were incubated with infected apoptotic HeLa cells before CD8 T-cell clones were added. After overnight incubation, cells were harvested and analyzed. A) Cytokine production by HIV-specific CD8 T cells on a representative sample. Among the lymphocyte population, CD8 T cells were gated and analyzed for IFN-γ, TNF-α, IL-2 and MIP-1β production. Cytokine production by HIV-specific CD8 T cells (B) or vaccinia-specific CD8 T cells (C) was determined. Virus variants are indicated on the x-axis; percentages CD8 T cells producing any cytokine are indicated on the y-axis. P-values between NYVAC-C and the mutants are indicated. Mean and standard deviation of four to six repetitions are shown. NYVAC-C elicited only very low numbers of cytokine-producing HIV- or vaccinia-specific CD8 T cells, only detected with the higher virus inoculum. Deletion of B19R from the parental NYVAC virus strain did not improve cytokine production. In contrast, moDCs cross presenting NYVAC-C-KC induced enhanced cytokine production by HIV- and vaccinia-specific CD8 T cells compared to NYVAC-C; additional deletion of the B19R gene in the NYVAC-C-KC background did not further increase cytokine production.

In addition to the effects of these virus mutants on DC maturation, we studied the functional differences between the different recombinant virus vectors *in vitro*. Towards that end, a cross-presentation assay was developed to determine the ability of moDCs to cross-present antigens from apoptotic infected HeLa cells. The gating strategy is shown in figure 7A; the total percentage of cytokine producing HIV- and vaccinia-specific CD8 T cells is indicated in figure 7B and C, respectively. MoDCs cross presenting NYVAC-C-KC induced enhanced cytokine production by HIV- and vaccinia-specific CD8 T cells compared to NYVAC-C (p<0.009 and p = 0.029, respectively). This was observed at all virus doses tested. In contrast, NYVAC-C elicited only very low numbers of cytokine-producing HIV- or vaccinia-specific CD8 T cells, which was only detected with the higher virus inoculum (MOI 1, 5). Deletion of B19R from the parental NYVAC virus strain did not improve cytokine production by the CD8 T cell clones that were assayed. As expected, deletion of the B19R gene in the NYVAC-C-KC background did not further increase cytokine production by HIV-specific CD8 T cells.

In these studies, direct presentation could be ruled out since infected HeLa cells were irradiated to induce apoptosis and kill residual virus. Furthermore, no cytokine production was observed by HIV-specific T cells after incubation with infected HeLa only (data not shown). As expected, since none of the T-cell clones used were restricted by the HLA alleles expressed by HeLa cells [45].

These data illustrate that the restoration of replication competence, reflected by Gag expression in HeLa cells (figure 6), correlates with the ability of moDCs to cross-present antigens to HIV-specific T cells *in vitro* (figure 7). In agreement, improved cross-presentation to vaccinia-specific CD8 T cells is also observed when replication competency in human cells is restored in the NYVAC vector background.

### Increased HIV memory T-cell proliferation after infection with replication-competent NYVAC

**Figure 8:**
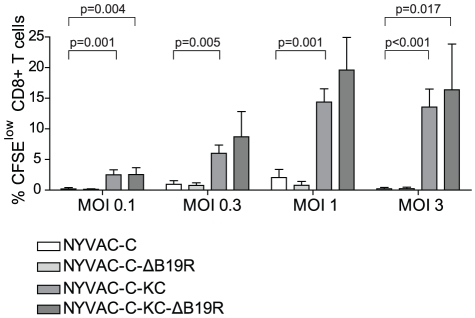
HIV-1-specific CD8 T-cell responses of unmodified and modified NYVAC using a CFSE proliferation assay. NYVAC vectors, either containing the HIV-1 clade C trangenes or empty, were evaluated *in vitro* using cryopreserved PBMCs from HIV-1-infected subjects. Cell proliferation using the CFSE dilution assay was measured 6 days after stimulation. At the end of the stimulation period, cells were stained for CD3, CD4, CD8 and a viability marker and analyzed by flow cytometry. Of note, NYVAC viruses were tested in a dose-dependent manner (ranging from 10^7^–10^4^ PFU, i.e. corresponding to a range of MOI going from 10-0.01). Shown is the proportion of proliferating cells (i.e. CFSElow cells) gated on live CD3+CD8+ T cells after 6 days of *in vitro* stimulation with the different doses of virus. Mean values, corrected for empty NYVAC background, and standard deviation of at least six experiments are shown. No proliferation of HIV-specific CD8 T cells was observed after infection with NYVAC-C and the B19R deletion mutant. In contrast, 15-20% CFSE^low^ CD8 T cells were present after NYVAC-C-KC or NYVAC-C-KC-ΔB19R infection, indicating increased proliferation after infection.

In addition to cytokine production by HIV-specific T-cell clones, the HIV-specific proliferative capacity of CFSE-labelled PBMCs from an HIV-infected long-term non-progressor was determined upon infection with the different viral vectors. Figure 8 represents CD8 T-cell proliferation as determined by CFSE dilution measured at day 6 after stimulation with the vectors in a dose-dependent manner. NYVAC-C-KC induced up to 15% CFSE^low^ CD8 T cells, indicating increased proliferation after infection. Increased proliferation was observed at multiple MOIs (p<0.032). Additional deletion of the B19R gene in the replication-competent vector did not significantly increase the proliferation of HIV-specific CD8 T cells. Surprisingly, NYVAC-C and the B19R deletion mutant were unable to induce any proliferation of CD8 T cells after infection (<5% CFSE^low^ CD8 T cells). NYVAC-wt and NYVAC-KC, both lacking the HIV-1 clade C transgenes, were unable to induce proliferation of the CD8 T cells (data not shown).

No effect of the gene deletion approach was observed in the NYVAC-C or NYVAC-C-KC background, indicating that only replication competence of NYVAC-C-KC resulted in enhanced proliferation of HIV-specific memory CD8 T cells. This is in agreement with the increased Gag expression in HeLa cells and moDCs as well as increased cytokine production by HIV-specific CD8 T-cells (figure 7).

## Discussion

To explore potential improvements of the immunogenicity of the NYVAC vector, we have used two strategies: deletion of a poxvirus gene known to encode a protein that may affect the immune response and development of attenuated replication-competent (in human cells) NYVAC. Of note, the replication-competent NYVAC vectors still maintain a highly attenuated phenotype, as assessed by mouse pathogenicity studies (Kibler et al., submitted for publication). To induce a broad T-cell response against HIV-1, the *env*, *gag*, *pol* and *nef* genes from a HIV-1 clade C isolate have been included in the viral vector [17]. The newly generated gene deletion and replication-competent NYVAC mutants have been extensively evaluated for their effect on gene expression and phenotype of DCs and their ability to stimulate CD8 T-cell responses *in vitro*.

In this manuscript, we show that deletion of the type I interferon binding protein B19R resulted in a NYVAC virus with enhanced IFN-α production, enhanced CD86 expression on cDCs and type-I IFN RNA expression. Most recently in the mouse, it has been shown that type-I IFN is the primary factor capable of eliciting DC maturation and consequently T-cell functions [46]. We also showed that restoration of replication competence of NYVAC, as in the NYVAC-C-KC variant, resulted in activation of pathways that enhance antigen processing and presentation and pathways associated with B-cell help (table 2). However, the replication-competent mutant did not elicit the induction of IFN genes and IFN-induced transcription factors. This is not surprising as the replication-competent mutant still expressed parental genes which inhibit IFN. Thus, we have generated a double mutant in which the B19R gene was deleted from the replication-competent virus to blunt the inhibitory effect of NYVAC-C-KC on IFN. Having both modifications thus resulted in IFN gene expression, activation of IFN-induced transcription factors, enhanced antigen processing and presentation, and induction of B-cell help, as observed in the gene expression profiles (summarized in table 2).

We have analyzed IFN-α production by the infected cDCs. cDCs produced IFN-α upon infection with NYVAC-C-ΔB19R, whereas no IFN-α production was detected upon infection of cDCs with NYVAC-C (figure 1). Accordingly, deletion of the B19R gene resulted in increased expression of IFN and IFN-induced genes and IFN-induced transcription factors in pDCs as well as cDCs (figure S1). This could be directly due to the absence of the soluble type-I IFN-binding protein. Type-I IFN, produced by the infected DCs, may subsequently lead to DC maturation. Indeed we observed a more mature phenotype of NYVAC-C-ΔB19R infected cDCs, reflected by the increased expression of CD86 compared to the parental NYVAC-C infected cDCs.

Interestingly, the deletion of B19R in the replication-competent virus induced IFN gene expression and the activation of IFN-induced transcription factors. However, these were at levels that were lower than the B19R mutant alone (figure 4 versus S2). Indeed, we were not able to detect IFN-α production by ELISA, which correlates with the absence of CD86 expression on cDCs after infection with NYVAC-C-KC-ΔB19R. In contrast, gene expression analysis showed that NYVAC-C-KC-ΔB19R presented the effects that were also observed with both single approaches; increased gene expression of IFN-induced genes and genes involved in antigen processing and presentation.

Infection with NYVAC-C-KC showed that pathways involved in viral replication process and viral infectious cycle were enriched, which confirms the replication capacity of the mutant virus in human primary cells, cDCs and pDCs. Indeed, enhanced HIV transgene expression was observed in moDCs, as well as HeLa cells, which in turn correlates with robust cytokine production by HIV-specific CD8 T cells in a cross-presentation assay. Although restoration of replication competence resulted in enhanced transgene expression, the expression levels differed between different human cell types (figure 6), possibly reflecting different kinetics in moDCs compared to HeLa cells. Since the HeLa cell line is a human papillomavirus infected cervical cancer immortal cell line, we expect primary human cells to behave like moDCs. Gag expression in NYVAC-C infected moDCs was very low, but increased upon infection with replication-competent NYVAC-C-KC. This is also supported by the increased expression levels of genes involved in the viral infectious life cycle in NYVAC-C-KC infected cDCs, in contrast to NYVAC-C infected cDCs (figure S2).

Gene array analysis showed numerous other gene expressions, quite apart from those mentioned above that are significantly up- or down-regulated indicating the effect of recombinant NYVAC in infected DCs. Since it is beyond the scope of this paper to extensively analyze and discuss all genes, we have not analyzed the functional relevance of these genes.

Since NYVAC also infects non-hematopoietic cells and the route of administration determines the cell types that are infected, cross-presentation probably plays a major role in the induction of vaccinia virus-induced CD8 T-cell responses [47], [48]. Therefore, we studied the ability to stimulate HIV- and vaccinia-specific CD8 T cells in a cross-presentation assay in which moDCs present antigens from apoptotic, infected HeLa cells. In accordance with increased Gag expression levels in NYVAC-C-KC infected HeLa cells, we observed high levels of cytokine producing HIV-specific CD8 T cells after incubation with cross-presenting moDCs. Moreover, increased cytokine production was also observed for vaccinia-specific CD8 T cells. In addition, infection of PBMCs from a long-term non-progressor with replication-competent viruses resulted in proliferation of HIV-specific memory CD8 T cells. Furthermore, gene array analysis showed improved antigen processing and presentation including enriched proteasome complex pathways, consistent with improved HIV-specific T-cell proliferation.

The replication-competent NYVAC-C-KC virus showed enhanced antigen expression and presentation to HIV- and vaccinia-specific CD8 T cells without inducing maturation of (cross-presenting) dendritic cells. These assays, however, have all been performed with either vaccinia- or HIV-specific T cells obtained from vaccinated or infected individuals, respectively, and were performed *in vitro*. The observations described in the present study with these clonal CD8 T cells were largely independent of costimulation. Costimulation is, however, important to prime T-cell responses *in vivo*.

Previously, Jackson et al. showed that deletion of the B19R gene from the vaccinia strain Wyeth had no effect on immunogenicity in mice [49]. In contrast, we here performed an in-depth analysis of infected DCs. The present study clearly shows that we were able to generate a phenotype in DC by selectively deletion or reinsertion of specific genes from the viral backbone. As described by Jackson et al., a single deletion of the B19R gene did not affect in vivo immunogenicity and therefore we combined the deletion with the restoration of replication competence. Unfortunately, the species specificity of the B19R protein might interfere with *in vivo* immunogenicity analysis in mice, such as performed by Jackson et al., thereby limiting the pre-clinical analysis. The *in vivo* immunogenicity of the combined recombinant NYVAC-C-KC-ΔB19R thus remains to be determined, for which the non-human primates is a suitable model.

The here described improved recombinant NYVAC vectors show potential applicability to HIV vaccination. Although the effect of the B19R deletion on type-I IFN production is clearly restrained by the introduction of replication competence, the NYVAC-C-KC-ΔB19R double mutant performs better as assessed by transcription profiling. This mutant shows improved expression of pathways enriched in IFN-induced genes and antigen processing and presentation pathways compared to the NYVAC-C-KC variant alone.

In conclusion, we have designed an improved candidate NYVAC-HIV vaccine. By restoring replication competence we were able to increase the expression of the transgene, which is important for the ability to induce robust T-cell responses *in vivo*. That enhanced transgene expression leads to enhanced cross-presentation to HIV- and vaccinia-specific T cells is expected from the observations of others [50]–[52] that level and stability of antigen expression are the two most important factors in the efficiency of cross-presentation and cross-priming.

## Materials and Methods

### Ethics statement

The Leiden University Medical Center, the University of Washington, and the Institute for Research in Biomedicine obtained written, informed consent from every blood donor in order to collect PBMC samples and approved the use of the material for this study. The study was approved by the institutional review board and by the ethics committee from the Centre Hospitalier Universitaire Vaudois and all patients gave written informed consent to use their material to make cell lines.

### Cells

Monocyte derived dendritic cells (moDCs) were obtained from cryopreserved or freshly isolated peripheral blood mononuclear cells (PBMCs) from buffy coats of healthy blood donors. CD14+ monocytes were isolated from PBMCs by positive selection with CD14 microbeads (Miltenyi Biotec). The obtained monocytes were plated at 1×10^6^ cells/ml and subsequently cultured with GM-CSF (800 U/ml) and IL-4 (500 U/ml) for 5 days to differentiate into moDCs as described previously [53]. Fresh medium containing GM-CSF and IL-4 was added at day 2.

Circulating conventional DCs (cDCs) and plasmacytoid DCs (pDCs) were obtained from freshly isolated PBMC by positive selection after staining with fluorescein isothiocyanate (FITC)-labeled anti-BDCA-1 (clone AD5-8E7) and phycoerythrin (PE)-labeled anti-BDCA-4 (clone AD5-17F6), respectively, followed by positive selection using anti-FITC or anti-PE microbeads (all from Milteny Biotec) and cell sorting. Purity of sorted DC populations was over 99%.

HeLa cells (ATCC) were cultured in IMDM containing 8% fetal bovine serum (PAA) and 80 IU/ml Natrium-penicillin (Astellas Pharma). Baby hamster kidney (BHK)-21 cells (ATCC) were grown in MEM plus 5% fetal bovine serum.

HIV-specific CD8 T cells were obtained from an HIV-1 seropositive long-term non-progressor. First, total PBMCs were depleted for CD4 T cells using CD4 dynabeads (Dynal) according to the manufacturer's protocol. The enriched CD8 T-cell population was subsequently stimulated with the specific peptide (5 µg/ml), irradiated HLA-matched PBMCs, 10% human T cell growth factor (TCGF, Zeptomatrix), human IL-15 (5 ng/ml, Tebu-bio), and 10% human AB serum. Specificity was confirmed after 4 weeks of culture. Although these CD8 T cells were not cloned from a limiting dilution, 99.8% of the T cells expressed the Vβ22 TCR, suggesting that these cells were obtained from a single precursor and can be considered clonal. Cells were restimulated every two weeks. Cells were left untreated for at least two weeks before use in antigen presentation assay.

Vaccinia-specific CD8 T cell clone CM.A2, derived from an HLA-A*0201 donor, was derived as described previously [54]. Clone CM.A2 was tested against a panel of known HLA-A*0201-restricted epitopes [55] and shown to be specific for WR082 18–26 (data not shown). Two other vaccinia-specific CD8 T cell clones were used and have shown similar results.

All cell cultures were kept at 37°C in a 5% CO_2_ incubator.

### Viruses

The generation of the recombinant NYVAC lacking the B19R gene or expressing the C7L and K1L genes is described elsewhere (Kibler et al., submitted for publication). The nomenclature and short description of the recombinant NYVAC variants is provided in table 1. Virological and pathogenic characterization of these vectors in cultured cells and in mice is described (Kibler et al., submitted for publication).

### Determining replication of viral vectors

Human HeLa cells or baby hamster kidney (BHK) cells were infected at a multiplicity of infection (MOI) of 5 with NYVAC-C, NYVAC-C-KC or NYVAC-C-KC-ΔB19R. Cultures were harvested immediately after infection or at 3, 12 and 24 hours post infection. Virus was released from cells by multiple rounds of freezing and thawing and titered on permissive BHK cells or BSC40 cells by plaque staining assays.

### HIV-1 Gag expression

The expression of Gag protein was measured in moDCs and HeLa cells at 6 and 24 hours after infection. To this end, cells were infected for one hour at MOI 1 and 5 and subsequently washed thoroughly. After 6 and 24 hours incubation, cells were harvested and Gag expression was determined by intracellular staining with an anti-Gag specific antibody (KC57, Beckman Coulter). Cells were analyzed on a FACSCalibur using CellQuest (BD). FACS data were analyzed with FlowJo (Tree Star, Inc.).

### Infection of cDCs and flow cytometry

cDCs or moDC obtained from freshly isolated PBMC were infected with the different viruses at three different MOIs (0.1, 0.3 and 1). After one hour of incubation, the cells were washed extensively and plated into 24-well plates. Supernatant was harvested at 24 and 48 hours post infection for detection of IFN-α. Forty-eight hours after infection, cells were harvested and fixed in 4% paraformaldehyde. Cells were subsequently incubated with α-CD86 PE-Cy5 (clone IT2.2), α-CD80 PE-Cy5 (clone 2D10.4), α-CD11c Alexa Fluor 700 (clone 3.9) (all from eBiosciences), α-CD40 APC, α-HLA-ABC FITC, α-HLA-DR PE, α-CD70 PE (all from Becton Dickinson). Cells were analyzed on a LSRII flow cytometer using DIVA (BD). FACS data were analyzed with FlowJo.

### IFN-α ELISA

Supernatant from infected DCs was harvested 48 hours post infection. IFN-α production was analyzed by ELISA (human IFN ELISA kit; PBL Interferonsource) according to the manufacturer's protocol.

### Antigen presentation assays

Antigen presentation to HIV- and vaccinia-specific CD8 T cells was studied using moDCs cross-presenting antigens from HeLa cells that were infected at different MOI. In addition, the cytokine production of HIV- and VACV-specific CD8 T cells was assessed.

For that, HeLa cells were harvested by EDTA and infected at different MOI for 1 hour. Cells were extensively washed to remove residual virus. After overnight incubation, cells were irradiated with UV-C (200 µW/cm^2^) to ensure that no residual virus and no viable cells were present and thus exclude direct presentation. Apoptotic virus-infected HeLa cells were harvested and added to moDCs at a 2∶1 ratio. After 6 hours incubation, HIV- or vaccinia-specific CD8 T cells were added (at approximately 5 T-cell: 1 DC ratio) followed by overnight culture at 37°C/5%CO_2_. Brefeldin A (10 µg/ml, Sigma-Aldrich) was added to retain cytokines within the T cells allowing the detection of multiple cytokines. After 18 hours, intracellular cytokine staining (ICS) was performed as described [56]. Cells were fixed and permeabilized using Cytofix/Cytoperm™ Fixation/Permeabilization Solution Kit (BD). Cells were then incubated with α-TNF PE-Cy7 (clone MAb11, eBiosciences), α-IFN-γ FITC, α-IL-2 APC, α-MIP-1β PE (all three from BD) and α-CD8 PerCP (Dako). After washing, cells were analyzed on a LSRII flow cytometer using DIVA (BD). FACS data were analyzed with FlowJo. Net accumulation is the percentage of live CD8+ cells expressing a specific cytokine upon stimulation with moDCs loaded with apoptotic virus-infected HeLa cells minus the percentage expressing the cytokine when NYVAC-wt infected HeLa were used. P-values were calculated using Mann-Whitney U test using SPSS 16.0 (SPSS Inc).

### *Ex vivo* proliferation assay

Overnight-rested cryo-preserved PBMCs were washed twice, resuspended at 1×10^6^/ml in PBS and incubated for 7′ at 37°C with 0.25 µM 5,6-carboxyfluorescein succinimidyl ester (CFSE, Molecular Probes, USA) as described [57]. Then, the reaction was quenched with one volume of FCS and cells were washed twice. Cells were then cultured (1×10^6^ in 1 ml of complete medium) in the presence of modified and unmodified NYVAC vectors at different MOIs (ranging from 0.01–10), medium alone (negative control) or Staphylococcal enterotoxin serotype B (SEB, 40 ng/ml, positive control). At day 6, cells were harvested, stained for dead cells using the Aqua LIVE/DEAD stain kit (Invitrogen) and then with CD3, CD4, CD8. After fixation, cells were acquired on an LSRII flow cytometer using DIVA (BD). FACS data were analyzed with FlowJo (8.8.2). The number of lymphocyte-gated events ranged between 1×10^5^ and 5×10^5^ in all experiments. P-values were calculated using Mann-Whitney U test using SPSS 16.0.

### Microarray data analysis

Infected cDCs and pDCs were harvested 6 hours post infection and the RNA was extracted using the RNeasy Mini Kit (Qiagen) according to the manufacturer's protocol. Quantification and quality control of extracted RNA was obtained as previously described [58]. Briefly, RNA quantification was performed using a spectrophotometer (NanoDrop Technologies) and RNA quality was assessed using the Experion automated electrophoresis system (Bio-Rad). Total RNA was then amplified and labeled using the Illumina TotalPrep RNA Amplification kit, which is based on the Eberwine amplification protocol [59]. The biotinylated cRNA was hybridized onto Illumina Human RefSeq-8 BeadChips V2 and V3 at 58°C for 20 hrs and quantified using Illumina BeadStation 500GX scanner and Illumina BeadStudio v3.

Illumina probe data were exported from BeadStudio as raw data and were screened for quality; samples failing chip visual inspection and control examination were removed. Probeset from the two Illumina platforms were mapped to a common probeset Id using a mapping file provided by Illumina. A dataset containing probeset common to both platforms was then used for subsequent steps. Gene expression data was preprocessed and analyzed using Bioconductor (www.bioconductor.org), an open-source software library for the analysis of genomic data based on R (version 2.9), a language and environment for statistical computing and graphics (www.r-project.org). The R software was used to quantile-normalized, and to minimum-replaced (a surrogate-replacement policy) values below background using the mean background value of the built-in Illumina probe controls as an alternative to background subtraction (which may introduce negative values) to reduce ‘over inflated’ expression ratios in subsequent steps. Bioconductor's genefilter package was used to filter out genes with low expression and insufficient variation in expression across all samples tested. Expression values retained after this filtering process presented intensities greater than 100 units in at least 2 samples and a log base 2 scale of at least 0.2 for the interquartile range (IQR) across all tested samples. The resulting matrix showing filtered probeset as rows and samples as columns was used as input for subsequent statistical analysis.

To identify differentially expressed genes, we used Bioconductor's “Linear models for microarray analysis” (LIMMA) [60] package which estimates the fold-change between DCs infected with different pox viruses by fitting a linear model and using an empirical Bayes method to moderate standard errors of the estimated log-fold changes for expression values from each gene. P values from the resulting comparison were adjusted for multiple testing according to the method of Benjamini and Hochberg [61]. This method controls the false discovery rate, which was set to 0.05.

To determine whether our expression data sets obtained from gene expression profiling of dendritic cells infected with different poxviruses are enriched in known biological pathways, we used Gene Set Enrichnment Analysis (GSEA), a non-parametric annotation-driven statistical analysis method. To evaluate the degree of enrichment the GSEA method calculates an Enrichment Score (ES) based on Kolmogorov-Smirnov statistics. We systematically tested gene sets from the Molecular signature Database (MsigDB, http://www.broad.mit.edu/gsea/msigdb) which are composed of (1) 1,892 gene sets (C2 collection) collected from different sources such as online known canonical and metabolic pathways and list of differentially expressed genes from publications available in PubMed, to which we added a collection of 28 immune related gene sets described by Chaussabel, et al. [43]; (2) 837 gene sets (C3 collection) that contain genes that share a cis-regulatory motif that is conserved across the human, mouse, rat, and dog genomes and represent known or likely regulatory elements in promoters and 3'- UTRs; (3) 1454 gene sets (C5 collection) that contain Gene Ontology terms. The statistical significance of a gene set's ES is estimated by an empirical genes-based permutation test procedure. To account for multiple hypotheses testing, GSEA normalizes the ES for each gene set to account for variation in set sizes and calculates a false discovery rate (FDR) corresponding to each normalized ES.

Microarray data are Minimum Information About a Microarray Experiment (MIAME)-compliant, and the raw data have been deposited in the Gene Expression Omnibus (GEO), accession number GSE26239.

## Supporting Information

Figure S1 GSEA of enriched pathways in NYVAC-C-ΔB19R infected DCs. GSEA of the list of genes ranked according to the expression difference between NYVAC-C and NYVAC-C-KC-ΔB19R in pDCs (A–C) and cDCs (D–E). GSEA using C2 database (A, D), C3 database (B, E) and C5 database (E) is shown. Figure shows the pattern of enrichment using selected significant pathways and their top 5 genes members selected from the leading edge subset (genes that contribute most to the enrichment score). The left gray and blue section of the figure shows the pathway membership for each gene (blue, present in the pathway; grey, absent). The heatmap shows the expression level of each gene scaled to have mean zero and standard deviation one (red, up-regulated; green, down-regulated). Each column in the heatmap represents a replicate (between 2 and 15). The genes indicated in the right vertical line represent some of the genes that are involved in the indicated pathways. The color key is depicted on the right side of the figure. NYVAC-C-ΔB19R induced the enhanced expression of genes in the type-I IFN-induced gene pathways and IL-1R in pDCs as well as cDCs.
(EPS)

Figure S2 GSEA of enriched pathways in NYVAC-C-KC infected DCs. GSEA of the list of genes ranked according to the expression difference between NYVAC-C and NYVAC-C-KC-ΔB19R in cDCs (A–B) and pDCs (C–D). GSEA using C2 database (A, C) and C3 database (B, D) is shown. Figure shows the pattern of enrichment using selected significant pathways and their top 5 genes members selected from the leading edge subset (genes that contribute most to the enrichment score). The left gray and blue section of the figure shows the pathway membership for each gene (blue, present in the pathway; grey, absent). The heatmap shows the expression level of each gene scaled to have mean zero and standard deviation one (red, up-regulated; green, down-regulated). Each column in the heatmap represents a replicate (between 12 and 18). The genes indicated in the right vertical line represent some of the genes that are involved in the indicated pathways. The color key is depicted on the right side of the figure.
(EPS)

Table S1 Genes uniquely upregulated in infected cDC after infection with NYVAC-C-KC.
(XLS)

Table S2 Genes uniquely upregulated in infected cDC after infection with NYVAC-C-KC-ΔB19R.
(XLS)

Table S3 Genes uniquely upregulated in infected cDC after infection with NYVAC-C-ΔB19R.
(XLS)

Table S4 Genes uniquely upregulated in infected pDC after infection with NYVAC-C-KC.
(XLS)

Table S5 Genes uniquely upregulated in infected pDC after infection with NYVAC-C-KC-ΔB19R.
(XLS)

Table S6 Genes uniquely upregulated in infected pDC after infection with NYVAC-C-ΔB19R.
(XLS)

Table S7 Common genes significantly up- or downregulated in DCs infected with either NYVAC-C-ΔB19R, NYVAC-C-KC or NYVAC-C-KC-ΔB19R.
(XLS)

Table S8 List of genes involved in the antigen processing and presentation pathway and B-cell function pathway.
(XLS)
